# Advancing age grading techniques for *Glossina morsitans morsitans*, vectors of African trypanosomiasis, through mid-infrared spectroscopy and machine learning

**DOI:** 10.1093/biomethods/bpae058

**Published:** 2024-08-17

**Authors:** Mauro Pazmiño-Betancourth, Ivan Casas Gómez-Uribarri, Karina Mondragon-Shem, Simon A Babayan, Francesco Baldini, Lee Rafuse Haines

**Affiliations:** School of Biodiversity, One Health and Veterinary Medicine, University of Glasgow, G12 8QQ, Glasgow, United Kingdom; School of Biodiversity, One Health and Veterinary Medicine, University of Glasgow, G12 8QQ, Glasgow, United Kingdom; Department of Vector Biology, Liverpool School of Tropical Medicine, L3 5QA, Liverpool, United Kingdom; School of Biodiversity, One Health and Veterinary Medicine, University of Glasgow, G12 8QQ, Glasgow, United Kingdom; School of Biodiversity, One Health and Veterinary Medicine, University of Glasgow, G12 8QQ, Glasgow, United Kingdom; Environmental Health, and Ecological Sciences Department, Ifakara Health Institute, Morogoro, Ifakara, P.O. Box 53, United Republic of Tanzania; Department of Vector Biology, Liverpool School of Tropical Medicine, L3 5QA, Liverpool, United Kingdom; Department of Biological Sciences, University of Notre Dame, 46556, Notre Dame, United States

## Abstract

Tsetse are the insects responsible for transmitting African trypanosomes, which cause sleeping sickness in humans and animal trypanosomiasis in wildlife and livestock. Knowing the age of these flies is important when assessing the effectiveness of vector control programs and modelling disease risk. Current methods to assess fly age are, however, labour-intensive, slow, and often inaccurate as skilled personnel are in short supply. Mid-infrared spectroscopy (MIRS), a fast and cost-effective tool to accurately estimate several biological traits of insects, offers a promising alternative. This is achieved by characterising the biochemical composition of the insect cuticle using infrared light coupled with machine–learning (ML) algorithms to estimate the traits of interest. We tested the performance of MIRS in estimating tsetse sex and age for the first-time using spectra obtained from their cuticle. We used 541 insectary-reared *Glossina m. morsitans* of two different age groups for males (5 and 7 weeks) and three age groups for females (3 days, 5 weeks, and 7 weeks). Spectra were collected from the head, thorax, and abdomen of each sample. ML models differentiated between male and female flies with a 96% accuracy and predicted the age group with 94% and 87% accuracy for males and females, respectively. The key infrared regions important for discriminating sex and age classification were characteristic of lipid and protein content. Our results support the use of MIRS as a rapid and accurate way to identify tsetse sex and age with minimal pre-processing. Further validation using wild-caught tsetse could pave the way for this technique to be implemented as a routine surveillance tool in vector control programmes.

## Introduction

Tsetse are blood-feeding flies that can transmit trypanosome parasites of human and animal concern [1]. There are two parasite species that cause Human African Trypanosomiasis (HAT), or sleeping sickness: *Trypanosoma brucei gambiense* and *Trypanosoma brucei rhodesiense* [2], and infected patients can die if they do not receive treatment [3, 4]. The promising decline of cases in endemic areas [5] in recent years is due to ongoing disease- and vector-control efforts, but continued support is critical to ensure the success of disease elimination programmes. However, Rhodesiense HAT (the more severe form) is still a concern due to livestock and wildlife forming part of its transmission cycle. Animal African trypanosomiasis (AAT) affects wildlife and domestic animals, causing 3 million cattle deaths/year with agricultural losses nearing US$5 billion/year [6]. Both female and male tsetse can transmit trypanosomes, but only adult flies older than 20 days post-emergence that have ingested blood from a parasite-infected host can be infectious [7, 8]. Tsetse age is therefore crucial for estimating transmission risk and the efficacy of vector-control programmes. Accurate age grading in the field is crucial for disease monitoring and evaluation operations [9]; and an effective vector control intervention, which does not target a specific age group, will reduce the average age of a tsetse population. For instance, if only young flies are caught while monitoring vector control, it suggests that new flies are still emerging in the area and control measures have reduced the older population (likely the ones transmitting disease). Conversely, if we capture older flies, it could mean one of two things: either the control measures are not performing as anticipated, or there is a possibility of fly reinfestation from nearby areas. Both scenarios signal that the current strategy needs to be reassessed and adjusted.

Tsetse age grading for female flies currently relies on performing a labour-intensive ovarian dissection, which requires the use of a microscope and an experienced dissector. Female tsetse give birth to a larva every 9 days [10] throughout life, and the four ovarioles develop in a specific, predictable sequence; as each egg descends into the uterus, it leaves behind a scar (named a ‘relic’) that can be microscopically identified [11]. No new relics are created after the fourth ovarian cycle, thus limiting the value of this method in flies older than 7 weeks [11]. Furthermore, factors such as nutritional stress [12], tsetse strain [13], and temperature [14] can affect the length of this 9-day process, and even with adjustments, the method can be imprecise [15]. Ovarian dissections are time consuming and need to be performed while the tsetse is still ‘fresh’, and tissues maintain their form. After death, flies quickly become dehydrated and age grading is no longer possible by this method [16]. This makes it difficult to process large numbers of flies when monitoring control interventions.

The current situation is worse for male tsetse, as there are no dependable methods for age-grading them. Wing fray analysis in either wild male [17] or female flies is unreliable as artefacts can be introduced through trapping protocols. Other approaches like tsetse eye pigment (pteridine) analysis [18] and gene expression [19] are too complex or costly for routine use in field settings. Thus, all current age-grading methods are either too imprecise, laborious, or expensive.

In related insect studies, mid-infrared spectroscopy (MIRS) has proven to be a versatile technique for determining mosquito age and species in both insectary-reared and field-collected mosquitoes [20–23]. MIRS quantifies the energy a molecule absorbs based on its molecular vibrations [24, 25]. As the surface of most insects is covered with a complex mixture of cuticular proteins, polysaccharides, waxes, and other lipids, this tool provides a way to discriminate between different samples [23, 25, 26]. The chemical composition of male and female cuticles, as well as different species-specific signatures, can be resolved alongside more transient aspects such as cuticular changes over time [9]. Scanning a dried insect sample with MIRS is rapid (1–2 min) [20], and when combined with the use of machine-learning (ML) algorithms, it provides a powerful toolbox for researchers rapidly to assess vector populations with minimum sample processing and high accuracy [21–23].

In this study, we use ML algorithms to estimate the age and sex from MIRS of different fly tissues collected from insectary-reared tsetse (*Glossina morsitans morsitans)* of known age and sex. We also identified the regions of the tsetse mid-infrared spectrum associated with age and sex, to investigate the biological basis of our model predictions.

## Materials and methods

### Tsetse rearing

An age-stratified colony of *Glossina morsitans morsitans* Westwood, established in 2004 at the Liverpool School of Tropical Medicine (LSTM), UK, was daily maintained under the following conditions: 26–28°C, 68%–78% humidity and a 12 h/12 h light/dark cycle. Tsetse were fed three times a week on sterile defibrinated horse blood (TCS Biosciences Ltd, Buckingham, UK) using a silicon membrane feeding system.

### Tsetse sampling strategy and desiccation

Young, unmated female flies were first collected from emerging pupal pots (male emergence is delayed, and male collection was timed after the females had emerged). Both teneral (unfed, newly emerged) female and male collections were isolated from each other to prevent potential cuticular contamination with contact sex pheromones (cuticular hydrocarbon) during mating [27].

We collected in total 354 female and 187 male teneral tsetse from the LSTM colony for analysis. At specific ages, tsetse were killed by placed them in a killing pot with a chloroform-soaked cotton on top of the pot, placed on a thin layer of cotton wool inside a 15 ml falcon tube half-filled with silica gel beads, sealed and then stored at 4°C until required. Desiccated tsetse were transferred to 96-well plates in preparation for shipping to the University of Glasgow. Upon analysis, dried flies were dissected into three sections: head, thorax, and abdomen using dissection tweezers.

### Infrared spectroscopy

Spectra from individual heads, thoraces, and abdomens were taken by Attenuated Total Reflection (ATR) FT-IR spectroscopy using a Bruker ALPHA II spectrometer equipped with a Globar lamp, a deuterated L-alanine-doped triglycene sulphate (DLaTGS) detector, a Potassium Bromide (KBr) beam splitter, and a diamond ATR accessory (Bruker Platinum ATR Unit A225). Twenty-four scans were collected at room temperature between 4000 and 400 cm^−1^, and with 4 cm^−1^ resolution per sample. Background measurements were performed every 30 min or when the CO_2_ band at 2400 cm^−1^started to appear. Moreover, when measuring the tsetse samples, we made efforts to avoid practices that might introduce sources of bias such as: always measuring first young and then old samples, or first females and then males. Low-quality spectra were discarded using a custom script designed for mosquito spectra which consisted of three filters: elimination of spectra with atmospheric intrusion (CO_2_ and water vapour) by assessing the smoothness of the region between 3500 and 3900 cm^−1^, low intensity spectra measuring the average absorbance of the plateau in the spectra between 400 and 500 cm^−1^, and distorted spectra caused by the anvil [20, 28].

### Machine-learning analysis

**Figure 1.**
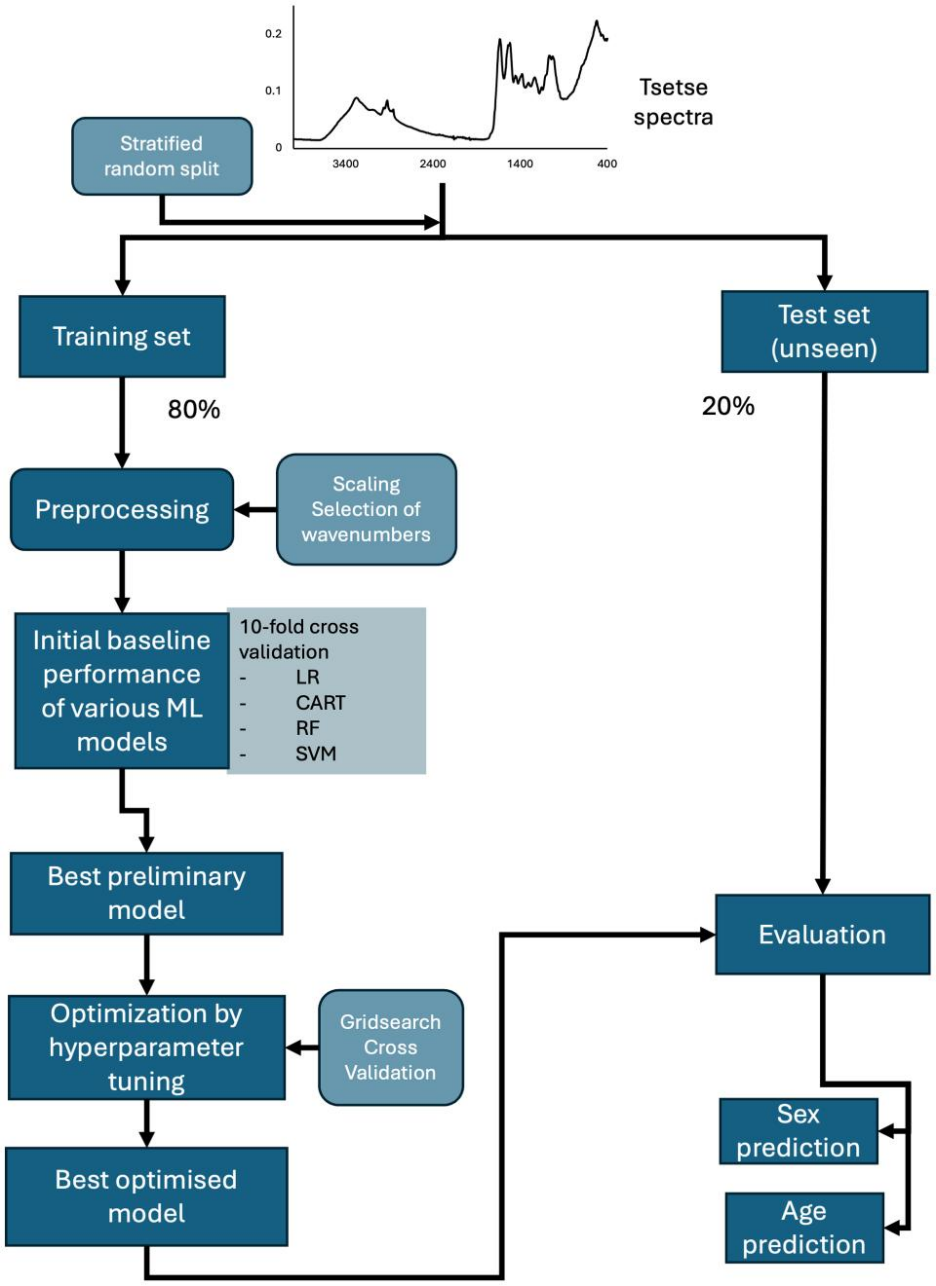
Flowchart of the analysis using supervised machine learning.

Uniform Manifold Approximation and Projection (UMAP) was applied for clustering analysis. Sex and age groups were binarized using one hot encoding [29]. First, we shuffled and split the dataset into the training (80%) and test sets (20%), stratified by sex and age groups (Supplementary Table S1). The training set was used to compute baseline performance of four ML algorithms: Logistic Regression (LR), Random Forest (RF), Support vector machine (SVC) with two kernels, and Classification and Regression Tree (CART) (using 10-fold cross validation and the default parameter settings on the training set). Additionally, a permutation score test was performed to evaluate if there was a dependency between the features (absorbance of each wavenumber) and classes (sex and age groups) (Supplementary Fig. S1). The best model was then optimized using hyperparameter tuning, which consists of choosing a set of optimal values for the model hyperparameters to maximize its performance. The remaining 20% of the data (the test set) were used for the final evaluation of the optimized models (Fig. 1). Model performance was measured by using confusion matrix and accuracy. ML was performed using Python 3.10 and scikit-learn 1.2.2.

## Results

### Optimization of tsetse desiccation

**Figure 2.**
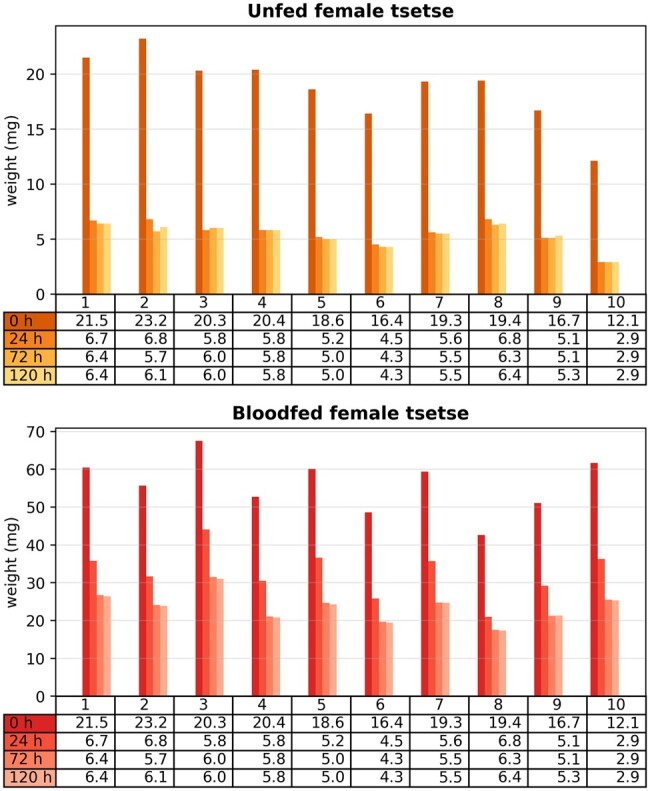
Desiccation time test for unfed and blood-fed female tsetse. Barplots show the weight of tsetse flies measured at different times of desiccation (0, 24, 72, 120 h).

To determine how long it took for tsetse in different nutritional states to dehydrate enough to reduce the noise in the spectra caused by water infrared absorption, we placed individual tsetse into 15 ml tubes containing a deep layer of silica gel under a thin cap of cotton wool. Fly weight loss was recorded daily until it stabilized. Unfed flies rapidly desiccated within 24 h, while fully engorged, blood-fed male and female flies took over 3 days to dehydrate the water-rich meal. Based on this data, we adopted a standardized ∼72 h of desiccation on silica for all flies subjected to MIRS analysis (Fig. 2).

### Differences between tsetse tissues

**Figure 3.**
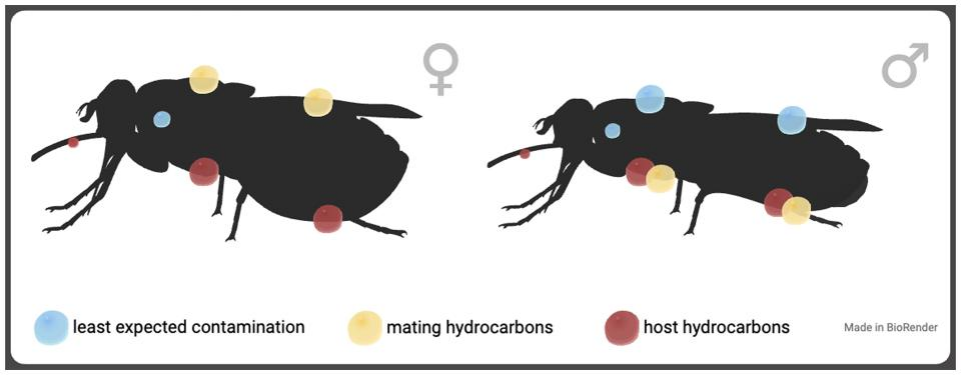
Tsetse biology and ecology suggest the heads and dorsal side of male tsetse or the lateral side of the thorax in both sexes would be the best areas (blue circles) to detect individual cuticular biochemical components.

Initial tests focused on finding the best body regions or tissues to give a high signal clarity when taking spectrometric readings, as the large size tsetse presented novel logistical challenges. Because wild-caught flies are likely to acquire foreign hydrocarbons from mating, blood feeding, or the resting environment, we sampled zones of the cuticle expected to show the least contamination (Fig. 3).

**Figure 4.**
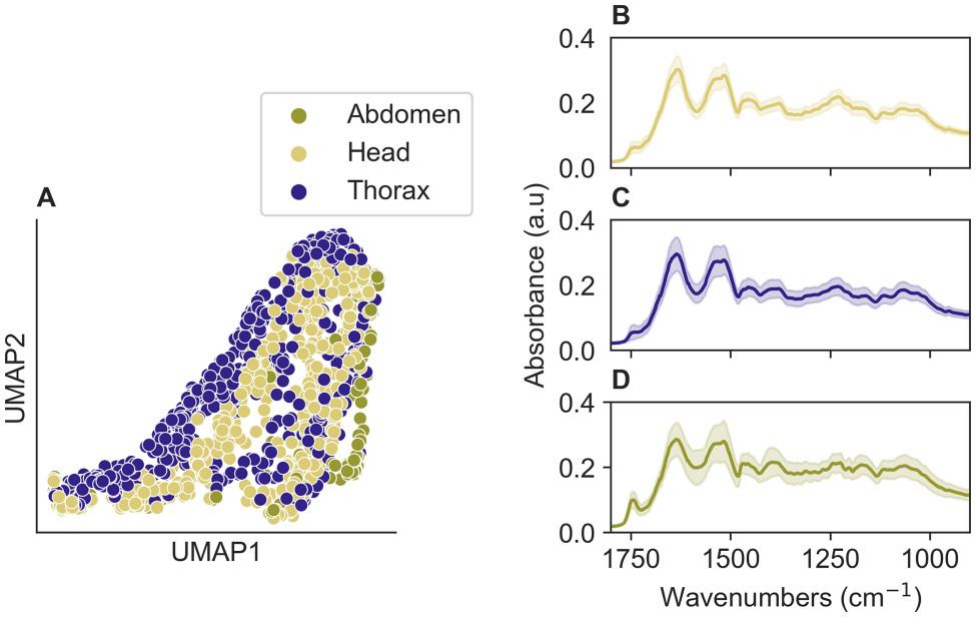
Spectra comparison from the head, thorax and abdomen. (**A**) Uniform Manifold Approximation and Projection (UMAP) of the abdomens, heads, and thoraces showed that the spectra collected from abdomens formed a separate cluster. Abdomens (olive green), head (yellow), thorax (purple). Comparing spectra from (**B**) head (yellow line) and (**C**) thorax (purple line) with (**D**) abdomens (olive green line) showed that the latter group had low intensity and great variability at specific wavelengths (primarily in the 1800 to 900 cm^−1^ region).

We further investigated the variation between spectra of different tissues. Spectra from fly abdomens differed substantially those from heads and thoraces (Fig. 4A), showing lower intensity and a higher variability, especially in the 1800 to 900 cm^−1^ region (Fig. 4B–D). Moreover, visual inspection of the abdomens indicated that despite ∼60 days in a sealed anhydrous environment, it was not possible to reach complete desiccation; consequently, water absorption noise was not eliminated, particularly if the fly had ingested a large blood volume prior to collection. This residual horse blood and water could be driving the greater variability of the abdominal spectra compared to the other tissues. In addition, previous work in other insects showed the thorax as a target tissue for MIRS [20, 21, 23]. Consequently, we decided to focus our analysis on the spectra obtained from heads and thoraces only. A total of 1071 spectra were therefore obtained by scanning the heads and lateral part of the thoraces of 541 flies of different ages (Table 1).

**Table 1.** Summary of aggregated samples sizes.

Sex	Age	Tissue	No. of spectra	No. of samples
**Female**	3 days	Head	133	136
		Thorax	136	
	5 weeks	Head	92	96
		Thorax	96	
	7 weeks	Head	120	122
		Thorax	122	
Male	5 weeks	Head	94	94
		Thorax	93	
	7 weeks	Head	93	93
		Thorax	92	
**Total number of samples**			**1071**	**541**

### Differences between sexes and age groups

**Figure 5.**
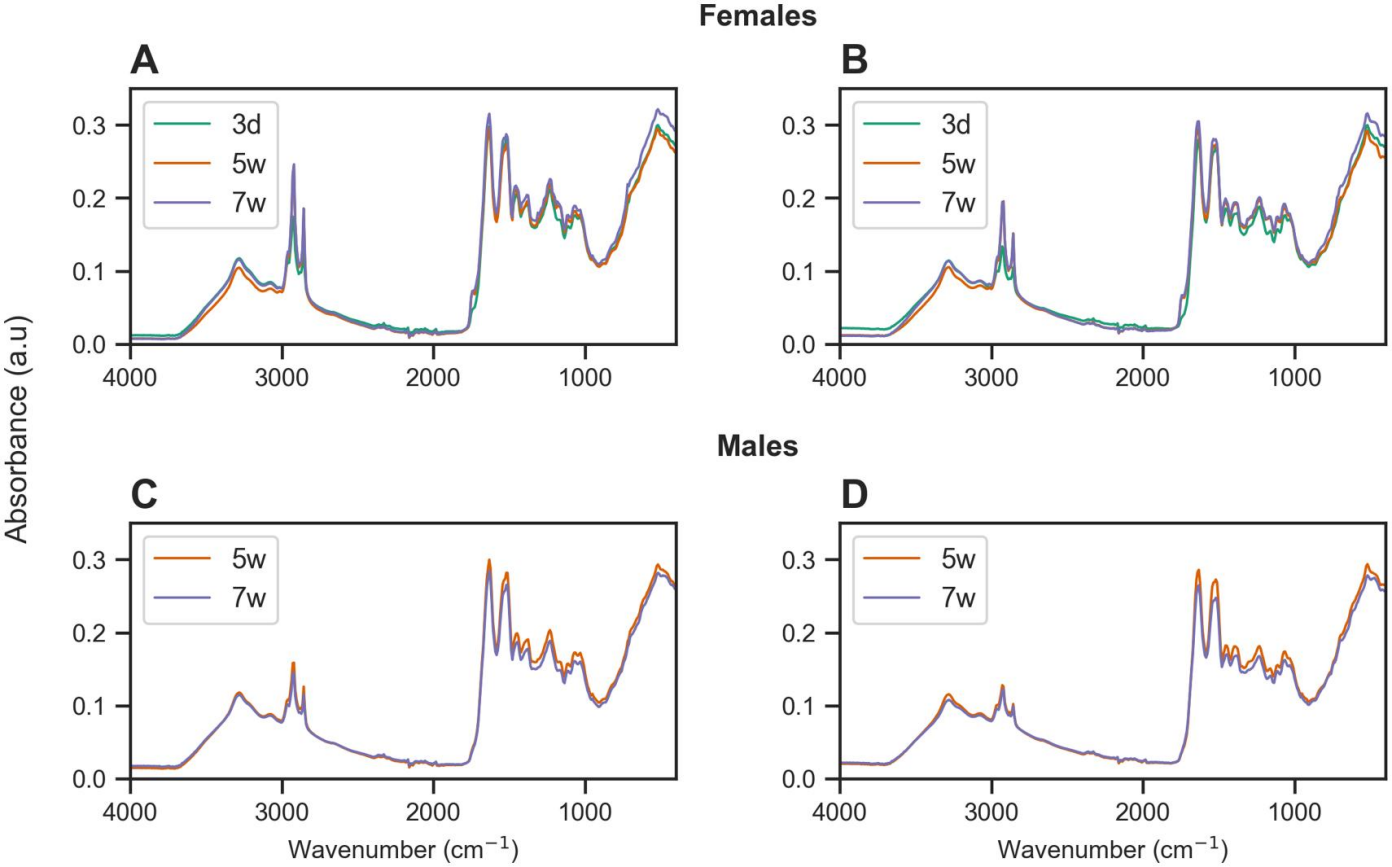
Mean spectra of females and males at different chronological ages (3 days = 3d; 5 weeks = 5w; 7 weeks = 7w) using different body parts (Heads: A, C. Thoraces: B, D).

**Figure 6.**
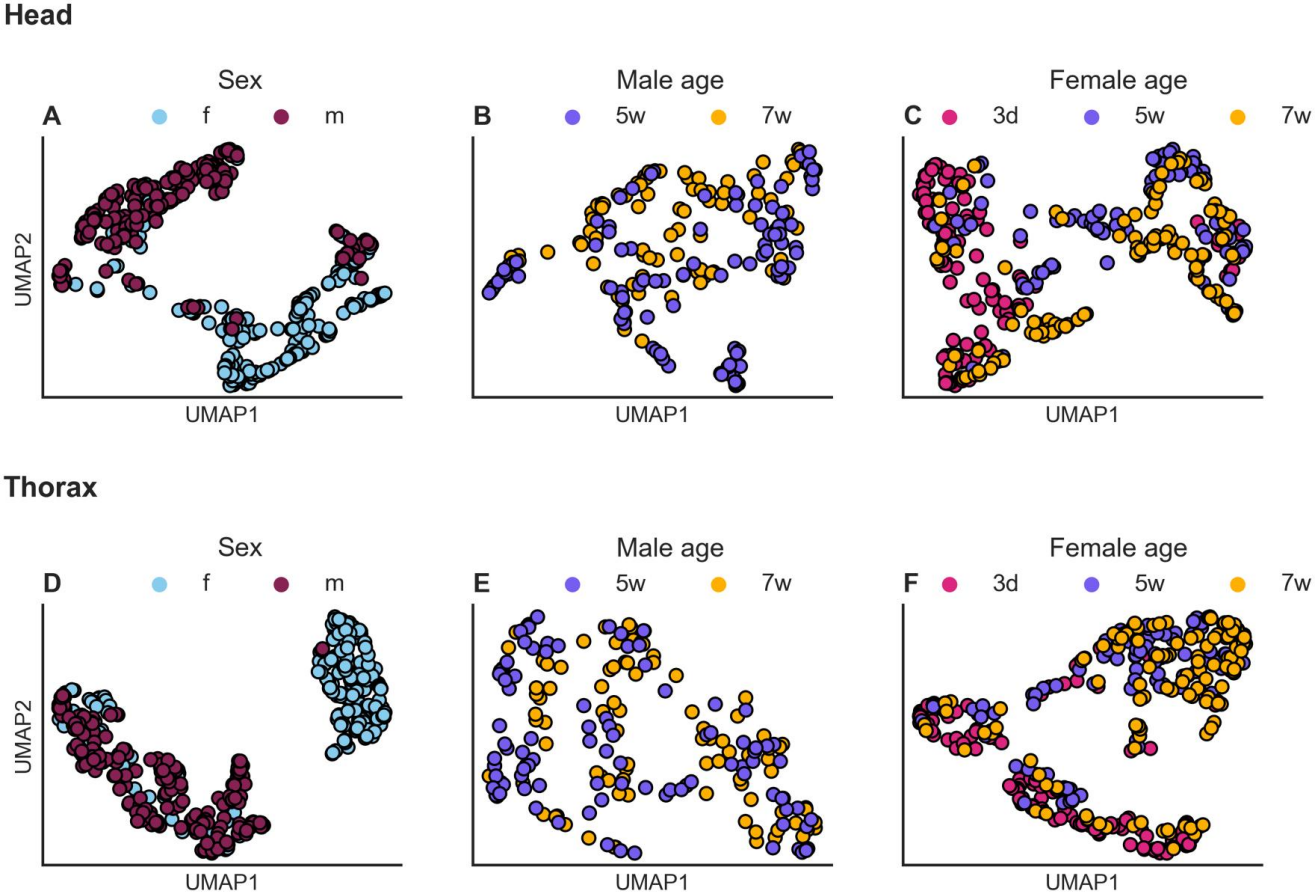
MIRS spectra according to tsetse sex and age from specific tissues. Unsupervised clustering of MIRS measurements using Uniform Manifold Approximation and Projection of MIRS in two-dimensional space using the heads and thorax. Samples are coloured by: (**A, D**) sex (females: blue, males: purple). (**B, E**) Males coloured by age (5 weeks: purple, 7 weeks: orange). (**C, F**) Females coloured by age (3 days: pink, 5 weeks: purple, 7 weeks: dark orange).

Mean spectra of head and thoraces of males and females at different chronological ages are shown in Fig. 5. We used the unsupervised ML algorithm UMAP to investigate whether the spectra from fly heads (Fig. 6A–C) and thoraces (Fig. 6D–F) differed between flies of different sex and age. Most of the male flies produced different spectra than females, with the thorax showing clearer clusters with fewer samples overlapping between them (Fig. 6A and D). For age groups, there were not clusters in males regardless the tissue (Fig. 6B and E). In females, there was a distinct cluster composed of old flies (5 and 7 weeks) when using the thorax. There was, however, a high overlap between samples from different age groups (Fig. 6C and F). These results show that MIRS reflects biochemical information associated with sex and age as expected from relative changes in the cuticular composition of tsetse.

### Sex and age prediction using the complete spectral data

**Figure 7.**
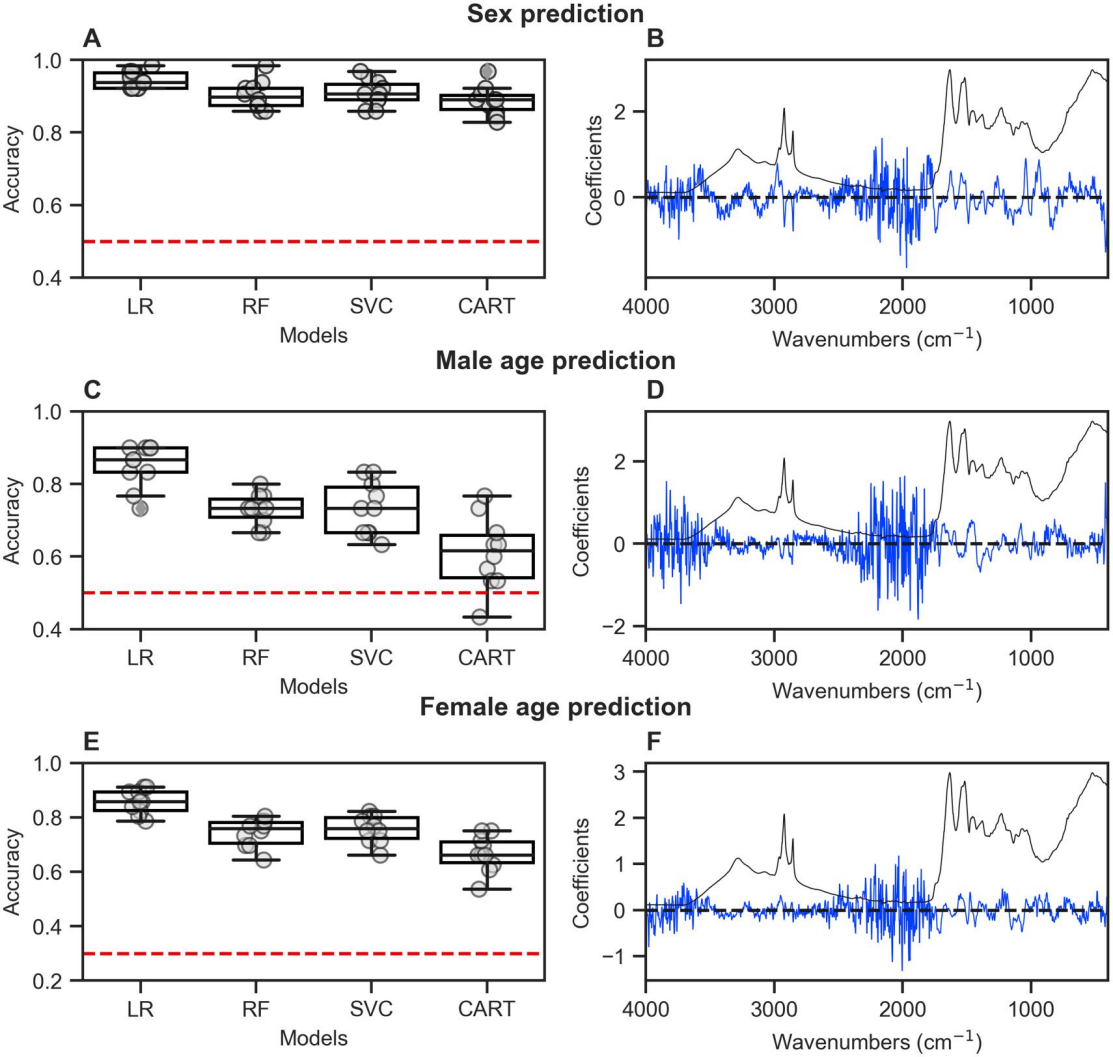
Prediction of tsetse sex and age using MIRS. Model performance on the training set of various ML models (LR: Logistic regression, RF: random forest, SVC: support vector machine and CART: decision tree classifier) for sex and age prediction using the heads of tsetse (**A, C, E**). Boxplots show the distribution of accuracies using 10-fold cross-validation. The horizontal dashed red line indicates a 0.5 accuracy for binary predictions (**A, C**) and 0.3 for a three-class prediction (**E**). Coefficients of the best model (blue line) plotted against the mean spectra of tsetse (**B, D, F**) show how the model relies on the 4000–3500 and 2500–1800 cm^−1^ regions for prediction, which are lacking key biological information.

To identify tsetse sex and age-specific patterns within our MIRS dataset, we compared LR, RF, SVC and the CART algorithms. Among these, LR had the highest accuracy (Fig. 7A) when estimating the sex of 5- and 7-week-old flies. Training accuracy was 94% when using both head (Fig. 7A) and thorax (Supplementary Table S2). Similar accuracies were obtained in the test set with 98.75% for the head, and 93.82% for the thorax (Supplementary Table S2). LR was also the most accurate algorithm for identifying age groups among flies of the same sex (Fig. 7C). For males, the thorax was marginally better at age prediction with accuracy of 88% compared to 85% for the head (Supplementary Table S2). Similar performance was found in the test set with 91.89% and 89.47% for thorax and head, respectively (Supplementary Table S2). In females, even though there was some difference in accuracy between the head and thorax on the training set (head = 86%, thorax = 92%), accuracy on the test set was similar for both tissues over 92% (Supplementary Table S2). To ensure generalizability of these models to other datasets including field flies, we analysed which wavenumbers (i.e. chemical bonds) contributed the most to the model predictions; we found that these were mostly based on flat regions of the spectra, between 4000–3750 cm^−1^ and 2250–1800 cm^−1^ (Fig. 7B, D and F), which are unlikely to contain biochemical information associated with insect cuticle [20] and are primarily used to monitor the presence of CO_2_ in the environment [24]. This phenomenon was observed with all predictive algorithms regardless of what tissue was used. To further investigate this, we applied the framework by Eid *et al*. [30]. Briefly, we divided the spectrum into three parts: two regions known to contain vibrations from key chemical bonds (3500–2500 cm^−1^ and 1800–600 cm^−1^ and one region where no chemical information associated with insect cuticle is expected (2500–1800 cm^−1^)). We then compared the accuracy of four algorithms: LR, SVM with two kernels (radial basis function and linear) and RF on each region. While the biochemical fingerprint regions (3500–2500 cm^−1^, 1800–600 cm^−1^) gave variable prediction accuracies (60%–96%), when using the region with no chemical information associated with insect cuticle (2500–1800 cm^−1^), two algorithms (LR and SVM with a linear kernel) could still predict different traits with high accuracy (83%–94%), indicating possible overfitting (Supplementary Table S3). To produce more generalizable models, we therefore chose to base our predictions on the spectral region of 1800–600 cm^−1^, which is known to contain the most relevant biochemical information in insects [21–23].

### Sex and age prediction using the biochemical fingerprint region of the spectra

**Figure 8.**
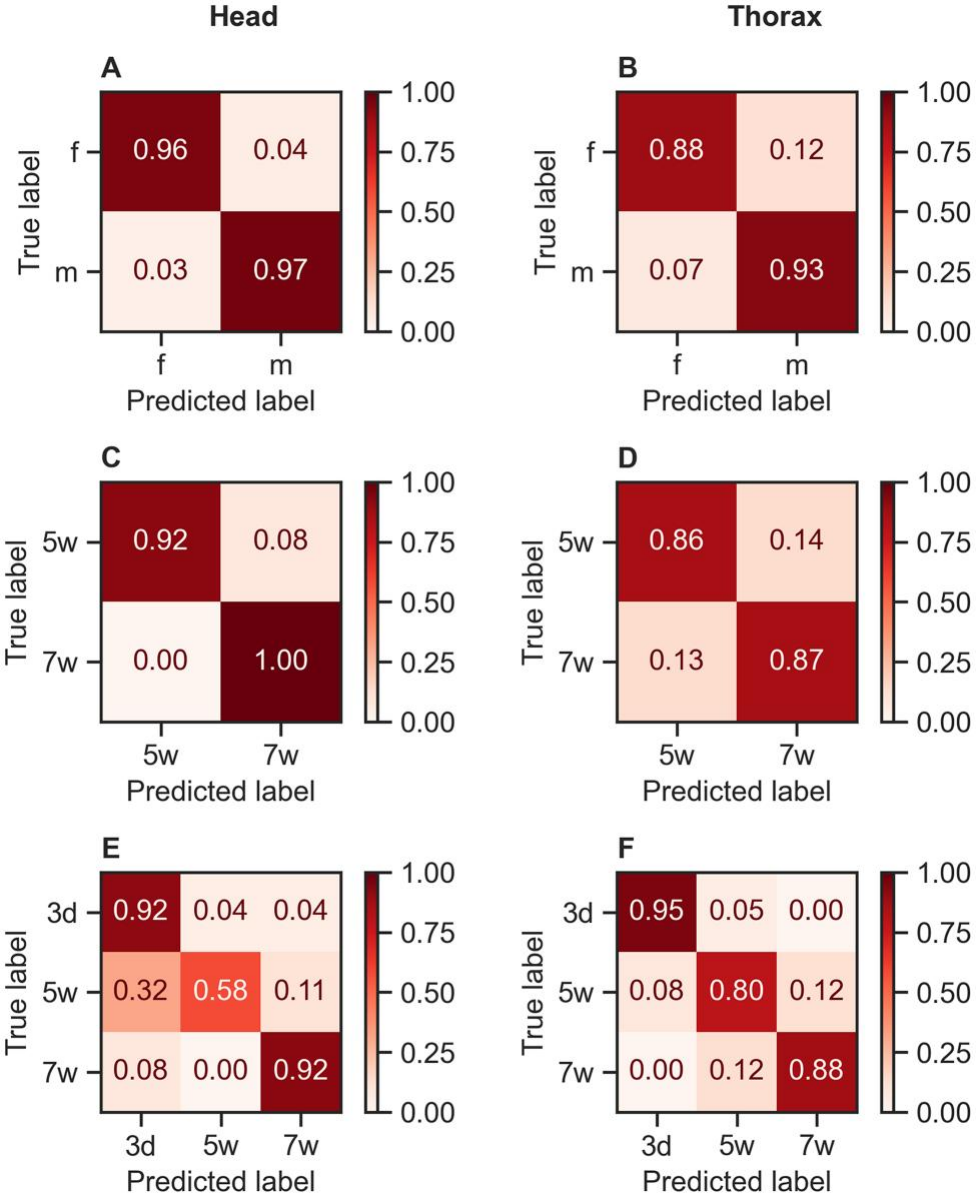
Confusion matrix for predicting tsetse sex and age using reduced number of wavenumbers. Accurate identification of females (f) and males (m) (**A, D**) and two-week age difference (5 weeks (5w) vs 7 weeks (7w) old) in male flies (**B, E**). Spectra from the thoraces of young female flies (3d post emergence) compared to older female flies (5 weeks (5w) and 7 weeks (7w) old) (**C, F**).

**Figure 9.**
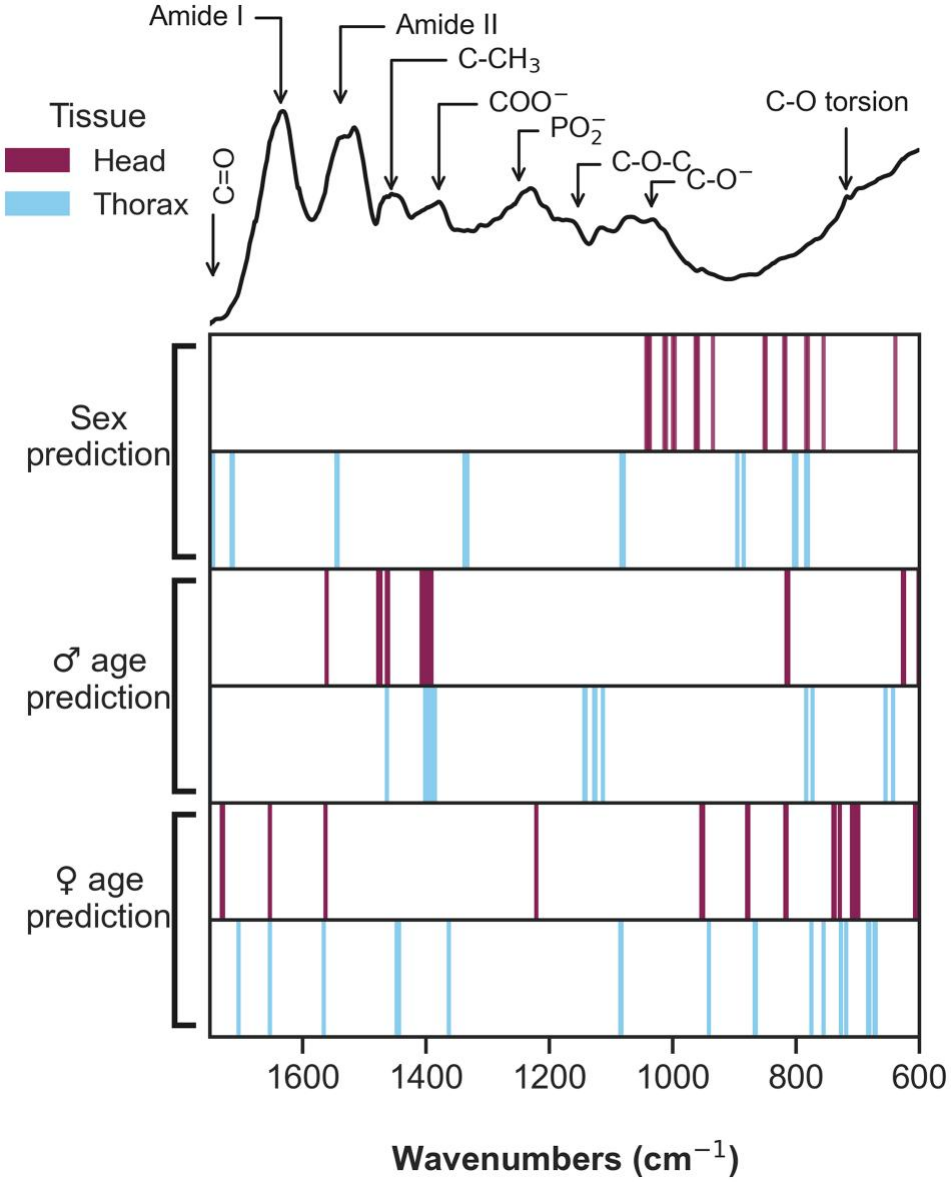
Important wavenumbers for predicting tsetse sex and age change depending on the trait predicted. Coloured lines represent the position of the most informative wavenumbers used by the models to predict sex, male age, and female age. Lines are coloured depending on the tissue used for MIRS: head (purple), thorax (light blue). Example spectra with band assignments are added on the top for reference.

**Figure 10.**
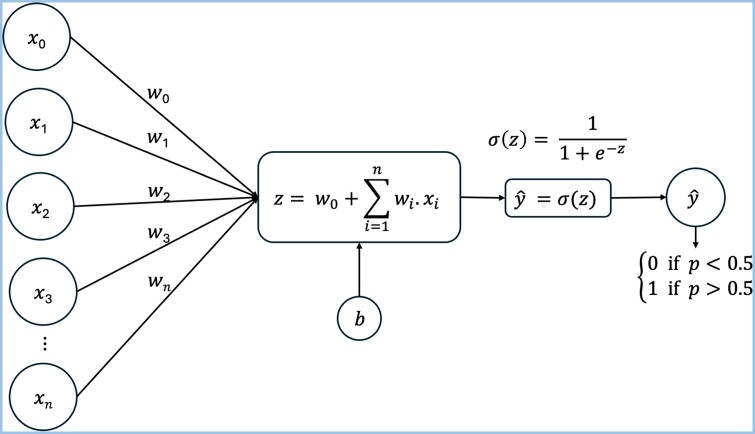
Flowchart of Logistic Regression algorithm.

When considering only the spectral region from 1750 to 600 cm^−1^, the accuracy of predicting fly sex and age marginally declined regardless of the algorithm used for analysis (Supplementary Fig. S2). The head was the tissue on which we obtained high accuracy in sex identification (Fig. 8A) while a higher proportion of females were misclassified as males when using the thorax (Fig. 8B). Accuracy in male age prediction was higher when using the head (Fig. 8C and D). Prediction of female age groups was high for very young and very old flies when using the head and thorax, but the model struggle to differentiate 5 and 7 weeks when using the head (Fig. 8E and F), which suggests that changes on MIRS signature is weaker in the head for older ages. Wavenumbers importance varied accordingly to the tissue and classification problem selected. Sex differences are mostly located in the region related to chitin for the head and lipids and proteins in the thorax (Fig. 9). For age prediction, the model used wavenumbers related to proteins (1636–1400 cm^−1^) and wax (800–600 cm^−1^) (Fig. 9). A summary of the performance and the flowchart of LR are shown in Table 2 and Fig. 10, respectively. Wavenumber importance and their assignments are presented in Supplementary Table S5, as well as the optimal hyperparameters (Supplementary Table S6). The same trend was observed when using additional preprocessing to the spectra (Savitzky-Golay second derivative) with a decrease in accuracy for age grading in both males and females (Supplementary Fig. S3 and Table S4). These results suggest that MIRS-ML is a promising approach when using the tsetse head or thorax to produce quality spectra reliably for sex and age prediction of laboratory-reared flies.

**Table 2.** Accuracy of tsetse sex and age prediction on males and females in the training and test set using Logistic Regression.

	Tissue	Accuracy (train set), %	Accuracy (test set), %
**Sex prediction**	Head	93.59 ± 2.38	96.25
	Thorax	92.46 ± 2.34	90.12
**Males age prediction**	Head	82.67 ± 4.67	94.74
	Thorax	83.67 ± 6.93	86.49
**Females age prediction**	Head	84.45 ± 3.27	82.61
	Thorax	84.73 ± 4.76	87.32

## Discussion

Here, we have shown for the first time how a MIRS + ML toolbox can be applied to predict the sex and age of desiccated insectary-reared tsetse. The spectra collected from the head and thorax, but not the abdomen, allow accurate sex prediction. Age grading was successful in both sexes, even when flies were only 2 weeks apart in age. When using exclusively the head, this toolbox can easily differentiate between females and males using the infrared region related to C–O stretch (chitin). UMAP projections are used as dimensionality reduction and visualization tool of high dimensional data that preserves its local and global structure [31]. We found that sex differences in spectra were identified on UMAP projection when using both tissues. However, the head showed less overlapping between the cluster, which explains the higher accuracy in sex prediction when using the head. It has been previously reported that *G. pallidipes* females possess a higher amount of cuticular lipids than males [32], which is likely linked to the female sex pheromone that constitutes the main cuticular hydrocarbon [32]. Considering this potential bias, we did not mix the two sexes for analysis since the signal difference can mask the differences between the ages of each sex.

When analysing the most discriminating regions for age grading in both males and females, some clear patterns emerged depending on the tissue and biological trait. In male flies, the C–CH_2_, COO^−^ bands were consistently important in age grading. However, the bands related to proteins, lipids, and the –(CH_2_)-rock functional group related to wax [24] were important across female tissues. Characterizing the informative and predominant wavenumbers is important for understanding the association between age and absorption bands, which can be used to optimize data collection [33] or model generalization [34]. An early staining method showed a relationship between cuticular layers in the thorax from laboratory and field caught flies [35]. Other methods using gene expression panels have also found that genes related to cuticular proteins were important for age grading. One study used RNAseq to analyse gene expression associated with age and sex in *G. m. morsitans* that were sourced from the same colony at LSTM [14]. Out of the ten genes shortlisted in the study, two proved to be sufficient for accurate age classification, one of these being cuticular protein 92F (GMOY002920). A second cuticular protein, 49Aa (GMOY005321), was also found on the list [19]. Previous work using MIRS with other insect vectors also reported differences in female cuticles between very young and old individuals, and the model predicted 3-day old females with minimal misclassification. However, when differentiating between 5- and 7-week-olds, the misclassification between both classes increased. These results are reflected on the UMAP projections, which showed how these two age groups clustered together compared to 3-day old samples. When using the thorax, there was an overlap of samples from 5- and 7-week-old, however, the very young samples remain in one cluster, contrary when using the head. These differences are reflected on the high accuracy for female age prediction when using the thorax.

When we used the complete spectra for training, we found that LR and SVM with a linear kernel used the region from 2500 to 1800 cm^−1^ to predict sex and age, which does not contain any biochemical information related to insect cuticles. To ensure the algorithms learn from the biochemical differences between sexes and age groups, we restricted the inputs to specific spectral regions and limited the features the model uses. The strength of ML lies in finding patterns to separate classes, but patterns can arise from confounding effects of contamination by water and CO_2_ rather than from the structural constituents of the specimen. It is important to diagnose and assess what the model is learning to rule out any bias and avoid overfitting. In spectroscopy data, variation between samples (i.e. baseline offset, variation on CO_2_ levels during different days when measuring) was robust enough for the model to classify age and sex accurately.

When determining the feasibility of using different tsetse tissues for analysis, the abdomen showed inconsistent spectra compared to the head and thorax, which might be caused by the presence of blood from previous meals and/or incomplete desiccation. However, the information from tsetse abdomens could still be used to identify blood meal sources, as demonstrated by the application of MIRS with *Anopheles* mosquitoes [36, 37].

In summary, our results provide proof-of-principle for how MIRS can detect cuticular signals linked to ageing in tsetse. Future validation of this technique using field samples is needed, where environmental cues (naturally minimized in housed insect colonies) impact ageing rates. The next step will be to test the MIRS toolbox against wild tsetse collected from endemic areas, and preferably a region currently implementing vector control strategies. The ML models we describe here need to be further refined using more insectary-reared flies alongside a small complementary set of field samples (age-graded when trapped) to be able to confirm the efficacy and accuracy of this method in the field [21].

## Conclusions

Our data strongly support the use of MIRS for high-accuracy age grading of both male and female *Glossina spp.* reared under insectary conditions. The method’s robustness, minimal maintenance, cost-effectiveness, and speed make it an ideal technique for vector surveillance programmes in resource-limited settings, and implementation will strengthen ongoing control efforts to prevent transmission of African trypanosomiasis.

## Supplementary Material

bpae058_Supplementary_Data

## Data Availability

The infrared spectral data generated for this study have been deposited in the Enlighten database and are available at http://dx.doi.org/10.5525/gla.researchdata.1564.

## Code availability

All codes to reproduce the machine learning analysis and figures are available at https://github.com/maurocolapso/Pazmino_TsetseMIRS_2023.git
